# Birth weight is associated with postmenopausal breast cancer risk in Swedish women

**DOI:** 10.1038/sj.bjc.6602203

**Published:** 2004-10-12

**Authors:** P H Lahmann, B Gullberg, H Olsson, H Boeing, G Berglund, L Lissner

**Affiliations:** 1 Department of Epidemiology, German Institute of Human Nutrition Potsdam-Rehbrücke, Arthur-Scheunert-Allee 114-116, 14558 Nuthetal, Germany; 2 Department of Social Medicine, Malmö University Hospital, Lund University, Malmö, Sweden; 3 Department of Oncology, Lund University Hospital, Lund University, Lund, Sweden; 4 Department of Internal Medicine, Malmö University Hospital, Lund University, Malmö, Sweden; 5 Department of Primary Health Care, Sahlgrenska Academy at Göteborg University and Nordic School of Public Health, Göteborg, Sweden

**Keywords:** birth weight, maternal hypertension, breast neoplasms, postmenopausal

## Abstract

There is some evidence that birth weight is associated with breast cancer. Whether this association differs between premenopausal and postmenopausal ages is still unclear. The results from this study suggest that higher birth weight is a risk factor for postmenopausal breast cancer (OR 1.06, CI 1.00–1.12, per 100 g), independent of selected early-life and adult factors.

High birth weight is a risk factor for breast cancer in some, but not all studies (reviewed by Potischman and Troisi, 1999; Okasha *et al*, 2003). Possible mechanisms for an association between larger size at birth and breast cancer include *in utero* exposure to high levels of oestrogens and growth hormones (Trichopoulos, 1990, 2003). The positive associations reported to date have been weak to modest and seem more apparent in premenopausal than in postmenopausal women (Potischman and Troisi, 1999; De Stavola *et al*, 2000; McCormack *et al*, 2003; Mellemkjaer *et al*, 2003). Findings from two recent studies suggest that the positive association between birth weight and breast cancer risk is present irrespective of age at diagnosis (Ahlgren *et al*, 2003; Kaijser *et al*, 2003).

The present study examines recorded birth weight in relation to breast cancer risk in Swedish women with postmenopausal breast cancer, controlling for other perinatal factors including socioeconomic status (SES) of origin and adulthood, as well as adult body mass index (BMI) measured prior to diagnosis.

## MATERIAL AND METHODS

This case–control study was nested within the Malmö Diet and Cancer (MDC) cohort study using available birth record data from 131 incident breast cancer cases diagnosed between 1991 and 2001 and 345 age-matched controls. The MDC-study, a collaborative centre of the European Prospective Investigation into Cancer and Nutrition (EPIC) (Riboli *et al*, 2002), comprises 17 035 female participants residing in Malmö, the third largest city in Sweden (Berglund *et al*, 1993).

The present analysis is restricted to breast cancer incidence in the period from study enrolment (1991–1996) until December 2001 among 5313 women born in Malmö between 1924 and 1950. We further restricted the analysis to 89 singleton female cases aged ⩾55 years at breast cancer diagnosis and age-matched controls without history of breast cancer (*n*=238), due to low statistical power for the analysis in women aged <55 years. Subjects' hospital delivery records were located in the city archive of Malmö using the civil registration number of the mother, which was available through record linkage to the subject. Cases were matched with controls by age (years) at entry of the MDC-study. We aimed at identifying three controls per case. Due to limited resources, and exclusion of twin births, some cases were matched 1 : 1 (*n*=11) or 1 : 2 (*n*=7). The breast cancer cases were identified by active follow-up and by record-linkage with regional and national cancer registries using individual civil registration numbers assigned to all residents in Sweden.

We abstracted birth characteristics as well as maternal information from the available birth records. Gestational age was estimated by using information on last menstrual period and delivery date. Unclassified gestational hypertension and/or proteinuria (Davey and MacGillivray, 1988) was used as proxy for pre-eclampsia, and is referred to as maternal hypertension/proteinuria in this study. Information on parental occupation, a marker of SES at origin, and adult characteristics, specifically BMI and educational level, were obtained from the database of the baseline examination (1991–1996). SES was classified according to the Nordic Occupation Classification System (Statistics Sweden, 1985). The MDC-study and the nested study were both approved by the Ethics Committee at Lund University, Sweden.

We used conditional logistic regression to examine the effect of birth weight on breast cancer risk, adjusting for other perinatal and later-life factors: gestational age (weeks), maternal hypertension/proteinuria (no/yes), birth year, parental occupation (low to high), BMI (kg m^−2^), educational level (low to high). Birth year was included in all analyses to adjust for cohort effects. Birth weight was modelled both as a categorical (<3000 g reference, 3000–3499 g, 3500–3999 g, ⩾4000 g, or <3000 g reference, ⩾3000 g) and continuous variable.

## RESULTS

On average, breast cancer cases had a 93.5 g higher birth weight than controls (Table 1

**Table 1:** Perinatal and adult characteristics by breast cancer status, Malmö Diet and Cancer Study (*n*=327)

**Characteristic**	**Controls (*n*=238)**	**Cases (*n*=89)**
	Mean (s.d.)	
Birth weight (g)	3380.6 (560.4)	3474.1 (572.0)
Gestational age (weeks)^a^	39.5 (1.9)	39.3 (2.1)
BMI (kg m^−2^) at baseline^b^	25.7 (4.5)	25.5 (4.3)
Age (years) at baseline^b^	58.3 (5.9)	58.0 (6.0)
Age (years) at diagnosis	—	63.0 (5.6)
		
	*N* (%)	
*Birth year*		
1924–1930	60 (25)	25 (28)
1931–1940	130 (55)	47 (53)
1941–1950	48 (20)	17 (19)
		
*Maternal hypertension/proteinuria*		
No	163 (68)	67 (75)
Yes	75 (32)	22 (25)
		
*Parental Occupation*^c^		
1 Low	49 (21)	14 (16)
2 Medium	84 (35)	35 (39)
3 High	27 (11)	10 (11)
4 Unknown	78 (33)	30 (34)
		
*Own educational attainment*		
1 (⩽8 years)	91 (38)	29 (33)
2 (9–12years)	129 (54)	53 (60)
3 (>12 years, university degree)	17 (7)	7 (8)

a Numbers of cases and controls do not add up to total study population due to missing data on gestational age (*n*=3).

b Start of follow-up (1991–1996).

c Low (unskilled manual worker), medium (skilled manual and low nonmanual worker), high (middle and high nonmanual worker), unknown (mixed group: farmers, employers, self-employed, missings).

). There were fewer cases with birth weights under 3000 g (15.7%) than controls (22.3%). Most subjects (93%) had been born after 37 completed weeks of gestation irrespective of case status. Mean gestational age in cases and controls were virtually identical. Maternal hypertension/proteinuria tended to be higher in controls than in cases. Neither mean maternal age nor birth order differed between cases and controls (not shown). The 89 cases had a mean age at diagnosis of 63.0 years (range 55–76).

Increased birth weight was significantly associated with elevated risk of postmenopausal breast cancer (Table 2

**Table 2:** Odds ratios (ORs) and 95% confidence intervals (CIs) for breast cancer by birth weight at postmenopausal ages (cases ⩾55 years at diagnosis), Malmö Diet and Cancer Study

**Birth weight (g)**	**Model 1 Adjusted for perinatal factors**			**Model 2 Adjusted for perinatal factors and parental occupation**		**Model 3 Further adjusted for adult factors**	
	**Cases^a^**	**OR**	**CI**	**OR**	**CI**	**OR**	**CI**
Birth weight (100 g)	88	1.06	1.00–1.12	1.06	1.00–1.12	1.06	1.00–1.12
		*P*=0.038		*P*=0.040		*P*=0.044	
*Categorical*							
<3000	14	Reference		Reference		Reference	
3000–3500	29	1.97	0.84–4.59	1.81	0.77–4.28	1.89	0.79–4.51
3500–4000	30	2.39	0.99–5.77	2.20	0.90–5.36	2.30	0.92–5.73
>4000	15	2.59	0.95–7.12	2.44	0.89–6.70	2.66	0.96–7.41
							
*Binary*							
<3000	14	Reference		Reference		Reference	
⩾3000	74	2.18	0.98–4.84	2.03	0.91–4.53	2.12	0.93–4.81

a Number of cases does not add up to total case population due to missing data on gestational age (*n*=1). Model 1: adjusted for gestational age, birth year, maternal hypertension/proteinuria. Model 2: adjusted for perinatal factors (model 1) and parental occupation. Model 3: adjusted for perinatal factors/parental occupation (model 2), and adult BMI, educational attainment.

). The risk increased by 6% per 100 g birth weight increase (OR 1.06, 1.00–1.12) adjusted for perinatal factors. Women who weighed over 4000 g at birth had a greater than two-fold excess risk (OR 2.59, CI 0.95–7.12) compared to controls (<3000 g) (model 1). Both gestational age and maternal hypertension/proteinuria were inversely, though not significantly, associated with breast cancer risk, being reduced by 44% (OR=0.56, CI 0.27–1.14). Neither the inclusion of parental occupation (model 2) nor the adjustment for adult risk factors, that is, BMI and educational level (model 3), attenuated the OR for continuous birth weight. Using percentage body fat as adult body measure instead of BMI did not appreciably attenuate the risk estimate for birth weight (data not shown). Similarly, multivariate adjustment for other breast cancer risk factors that were not included in model 3, such as age at menarche, parity, age at first birth, use of hormone replacement therapy (HRT), and usual alcohol consumption, attenuated the risk estimate by only 5%.

Birth weight, adjusted for gestational age, was positively correlated with adult BMI (partial *r*=0.07, *P*=0.18), which was itself not a significant risk factor for breast cancer in this study. Other indicators of birth size, such as birth length, ponderal index (g cm^−3^), and head circumference were positively, but not significantly, associated with postmenopausal breast cancer (data not shown).

## DISCUSSION

In this nested case–control study, increasing birth weight was associated with increased risk of postmenopausal breast cancer diagnosed at age 55 years or older, significantly so when birth weight was examined as continuous variable (6% risk increase per 100 g), and persisted after adjustment for other perinatal and adult factors.

Our finding in postmenopausal women may be contrasted with previous studies on premenopausal women. Case–control (Michels *et al*, 1996; Sanderson *et al*, 1996; Innes *et al*, 2000; Mellemkjaer *et al*, 2003) and prospective studies (De Stavola *et al*, 2000; McCormack *et al*, 2003) have generally yielded positive associations between high birth weight and early-onset breast cancer. However, birth weight was not found to be related to premenopausal breast cancer risk in Chinese women, considered a low-risk population (Sanderson *et al*, 2002). Moreover, Kaijser *et al* (2003) investigating a Swedish cohort of prematurely born women, found a positive association between birth weight and adult breast cancer risk independent of age at onset.

Of the five studies of birth weight and postmenopausal breast cancer (⩾50 years), only one, conducted in Denmark, indicated a significant positive trend in rates of breast cancer with birth weight (9% per 1000 g) (Ahlgren *et al*, 2003). A weak J-shaped pattern was noted in US women aged 50–79 years with the highest risk in the birth weight category >4500 g (OR 1.18, CI 0.92–1.51, *vs* reference group) (Titus-Ernstoff *et al*, 2002). In the other three studies, nonsignificant positive (Michels *et al*, 1996) and inverse (Sanderson *et al*, 1996; McCormack *et al*, 2003) trends were observed.

Strengths of our study were the availability of data on recorded birth weight as well as gestational age, an important determinant of foetal growth. Therefore data were not subject to recall bias or residual confounding due to lack of adjustment for gestational duration. Only one other study had information on both recorded birth weight and gestational age (McCormack *et al*, 2003).

Other perinatal factors that correlate with oestrogen level during pregnancy, particularly twinning, pre-eclampsia, maternal age, and birth order are considered early life risk factors for cancer (Ekbom, 1998; Potischman and Troisi, 1999; Okasha *et al*, 2003). Twin births were excluded from our study. Maternal hypertension/proteinuria was inversely associated with breast cancer, which appears to be in line with earlier Swedish reports indicating that pre-eclampsia reduced breast cancer risk in female offspring (Ekbom *et al*, 1992, 1997). Maternal age and birth order were not associated with breast cancer in this study (data not shown), and were omitted from the analysis since these factors did not significantly improve the tested models.

Data on birth outcome may be confounded by socioeconomic factors (Joseph and Kramer, 1996). However, in our study, adjustment for parental occupation did not affect the relation between birth weight and cancer risk, corroborating other reports using father's occupation as marker of early life SES (De Stavola *et al*, 2000; Vatten *et al*, 2002). In the few studies taking account of adult risk factors, including adult body measures, risk estimates of birth weight were not appreciably altered (Michels *et al*, 1996; De Stavola *et al*, 2000). Similarly, adjustment in our study for adult relative weight (BMI) did not change the magnitude of the effect of birth weight on breast cancer.

Despite lack of statistical significance in some of the modelling, there is a strong suggestion of an increase in breast cancer risk with higher birth weight in this population of Swedish postmenopausal women, independent of adult body size. This finding lends support to the importance of prenatal factors in the aetiology of breast cancer.
